# Human umbilical cord blood-derived MSCs trans-differentiate into endometrial cells and regulate Th17/Treg balance through NF-κB signaling in rabbit intrauterine adhesions endometrium

**DOI:** 10.1186/s13287-022-02990-1

**Published:** 2022-07-15

**Authors:** Qing Hua, Yong Zhang, Hongjuan Li, Haoran Li, Ranran Jin, Li Li, Yuancui Xiang, Meng Tian, Jingjing Wang, Lei Sun, Yali Wang

**Affiliations:** 1 Department of Obstetrics and Gynecology, Zhengzhou Central Hospital Affiliated to Zhengzhou University, Zhengzhou, Henan People’s Republic of China; 2 Branch Center of Advanced Medical Research Center, Zhengzhou Central Hospital Affiliated to Zhengzhou University, Zhengzhou, Henan People’s Republic of China; 3 Department of Translational Medicine Center, Zhengzhou Central Hospital Affiliated to Zhengzhou University, Zhengzhou, Henan People’s Republic of China

**Keywords:** Intrauterine adhesion (IUA), Human umbilical cord blood-derived mesenchymal stem cells (hUCB-MSCs), scRNA-seq, Trans-differentiation, NF-κB signaling

## Abstract

**Purpose:**

The fundamental cause of intrauterine adhesions (IUAs) is the destruction and reduction in stem cells in endometrial basal layer, resulting in endometrial reconstruction very difficult. The purpose of this study was to investigate the effects and underlying mechanism of human umbilical cord blood-derived mesenchymal stem cells (hUCB-MSCs) on the endometrial reconstruction after transplantation.

**Methods:**

hUCB-MSCs were isolated and identified by flow cytometry, osteogenic, adipogenic and chondrogenic differentiation assays. The rabbit IUA models were established and set five groups (control, 14/28th day after surgery, estrogen and hUCB-MSCs treatment). The number of endometrial glands and the fibrosis rate were evaluated using HE and Masson staining, respectively. Endometrial proliferation, angiogenesis and inflammation were evaluated by immunohistochemical staining of ER, Ki-67and TGF-β1, respectively. Single-cell RNA sequencing (scRNA-seq) was applied to explore the cell differentiation trajectory after hUCB-MSCs transplanted into IUA endometrium. Finally, molecular mechanism of hUCB-MSCs repairing damaged endometrium was investigated by RNA sequencing, qRT-PCR and Western blot assays.

**Results:**

After transplantation of the hUCB-MSCs, the increase in endometrial gland number, estrogen receptor (ER) and Ki-67 expression, and the decrease in fibrosis rate and TGF-β expression (*P* < 0.05), suggested the endometrial repair, angiogenesis and inflammatory suppression. The therapeutic effect of hUCB-MSCs was significantly improved compared with 28th day after surgery and estrogen group. ScRNA-seq demonstrated that the transplanted hUCB-MSCs can trans-differentiate into endometrial cells: epithelial, fibroblast and macrophage. RNA sequencing of six IUA samples combined with qRT-PCR and Western blot assays further revealed that hUCB-MSCs may regulate Th17/Treg balance through NF-κB signaling, thus inhibiting the immune response of damaged endometrium.

**Conclusions:**

Our study demonstrated that hUCB-MSCs can repair damaged endometrium through trans-differentiation, immunomodulatory capacities and NF-κB signaling, suggesting the treatment value of hUCB-MSCs in IUA.

**Supplementary Information:**

The online version contains supplementary material available at 10.1186/s13287-022-02990-1.

## Introduction

In women of reproductive age, severe injuries to the endometrium are often accompanied by intrauterine adhesion (IUA), which can result in menstrual disorders, dysmenorrheal, amenorrhea, recurrent spontaneous abortion and infertility [1, 2]. Currently, the gold standard treatment for IUA is hysteroscopic resection of the adhesions [3], followed by estrogen therapy to stimulate regeneration of the endometrium [4]. This treatment strategy is useful for patients with mild-or-moderate disease severity but not effective for severe IUAs [5]. The recurrence rate of severe IUA is up to 62.5% [1, 3], and the postoperative pregnancy rate is only 33.3% [6]. Current treatments can only temporarily restore the anatomy of the uterine, but cannot restore the structure and function of the endometrium. Due to impaired revascularization and ischemia in severe IUA [7], the clinical effects of these medications that promote blood perfusion such as aspirin and granulocyte colony-stimulating factor are limited [8, 9].

The cellular and molecular pathogenesis of IUA remains obscure and controversial. Some studies suggested that fibroblasts are activated by CTGF (connective tissue growth factor) through TGF-β after endometrial injury and then produce excessive fibrillar collagen, which inhibit the normal regeneration of endometrial mesenchymal stem cells [10, 11]. Studies also found that the expression of NF-κB in IUA endometrium was significantly higher than that in normal endometrium, which can promote the occurrence of endometrial inflammation [12]. Besides, damaged endometrial cells can promote Th2 immune response to promote fibrosis [13]. Thus, these cellular changes and molecular pathways may provide potential clues for new treatment of IUA to reverse inflammation and promote endometrial regeneration.

Although many methods have been used to treat severe IUA, the high recurrence rate and endometrial thinning limit the therapeutic effect. Therefore, how to regenerate the damaged endometrium and restore its function is an important problem and great challenge for IUA treatment. Recently, cell therapy for IUA is promising in the treatment of endometrial dysfunction [14]. Mesenchymal stem cells (MSCs) are considered to be an ideal cell type for tissue regeneration due to their high potential differentiation, self-renewal and immune regulation [15]. At present, the animal and clinical trials of MSCs in IUA treatment have been abundantly carried out, showing the ability of obvious endometrial repair and regeneration for MSCs [16]. Although MSCs have demonstrated to be a promising cell therapy in IUA treatment, these studies are still in the experimental research stage, and its advantages over estrogen therapy and the exact mechanism of treatment are worthy of further investigation.

Cord blood contains both hematopoietic and non-hematopoietic cells, of which mesenchymal stem cells are a very small percentage of non-hematopoietic cells. They can be considered as “very young” and have the potential for multilineage differentiation [17]. Human umbilical cord blood-derived MSCs (hUCB-MSCs) have been regarded as a favorable source for cell-based therapies because of their easy collection, low immunogenicity, and high proliferative potential. hUCB-MSCs have been used in the treatment of type I diabetes [18], osteoarthritis [19], acute graft-versus-host disease [20], etc. However, few studies have reported the application of hUCB-MSCs in endometrial repair.

In the present study, we used hUCB-MSCs to treat rabbit IUA model and explored the underlying therapeutic mechanisms. We used scRNA-seq to determine whether hUCB-MSCs transplanted into endometrium differentiate into endometrial cells and used RNA sequencing to investigate the role of immunoregulation and signaling pathway during hUCB-MSCs transplantation.

## Materials and methods

### Ethics

This study was approved by the Ethics Committee of Zhengzhou Central Hospital Affiliated to Zhengzhou University (ethics number: 202126). All experimental procedures in rabbits were conformed with the Guide for the Care and Use of Laboratory Animals of the National Institutes of Health (NIH Publication No. 85-23, revised 1996). Sexually mature female New Zealand Rabbit (2800–3000 g) at the age of 14–16 weeks were purchased from Pizhou Dongfang Breeding Co., Ltd. (permit number: SCXK (su) 2017-0002), Jiangsu, China. All rabbits were fed rabbit chow and water ad libitum under temperature-controlled environment at 25 °C with a 12-h light and dark cycle for 1 week.

### Isolation of hUCB-MSCs

Umbilical cord blood samples (about 50 mL each) with anticoagulant (EDTA) were collected from umbilical cord vein. Mononuclear cells (MNCs) were isolated by density gradient centrifugation for 30 min at 400 g and washed 3 times in PBS (Beyotime). The isolated MNCs were plated in 75cm^2^ cell culture flask containing mesenchymal stem cell basal medium (Yocon, China) and incubated at 37 °C in a humidified atmosphere of 5% CO_2_. Around the tenth day of culture, we can see adherent cells, while non-adherent cells were removed, and then the medium was changed every 3 days. After expansion to 80–90% confluence, the cells were harvested by 0.25% trypsinization and subcultured for further experiments. Inverted microscope was used to analyze the morphology of hUCB-MSCs. According to the International Society for Cellular Therapy, MSCs have three characteristics: (1) plastic adherent, (2) express CD105, CD73 and CD90 and not express CD45, CD34, CD14, CD11b, CD79α, CD19 and HLA-DR surface antigen, (3) differentiate into osteoblasts, adipocytes and chondroblasts in vitro [21]. Thus, hUCB-MSCs were analyzed by flow cytometry, osteogenic, adipogenic and chondrogenic differentiation assays.

### Flow cytometric analysis

Detached hUCB-MSCs were washed twice with ice-cold PBS, centrifuged and fixed in 4% paraformaldehyde. Then, cells were incubated with mouse anti-human CD45-FITC, CD34-FITC, CD11b-FITC, CD19-FITC, CD29-FITC, CD73-PE, CD105-APC-A750, CD90-APC and HLA-DR-FITC (all from BioLegend) in dark for 20 min. Cells were analyzed by flow cytometer (Beckman Coulter GmbH, Krefeld, Germany).

### Osteogenic, adipogenic and chondrogenic differentiation

To investigate the osteogenic and adipogenic differentiation potential of hUCB-MSCs, third-passage cells were plated at a concentration of 3 × 10^3^cells/cm^2^ and cultured with osteogenic and adipogenic medium (Sigma-Aldrich) for 3 weeks with medium changes twice weekly, respectively. At the end of differentiation, cells were stained with Alizarin Red S and Oil Red O, respectively. To induce chondrogenic differentiation, we cultured 3 × 10^5^ hUCB-MSCs/well in chondrogenic medium (Procell) for 4 weeks. Medium changes were carried out twice weekly, and chondrogenesis was assessed at 2–3-day intervals. Cells were fixed in 4% formaldehyde, dehydrated in an ethanol series and embedded in paraffin blocks. Blocks were cut, and sections were stained with Alcian Blue to evaluate chondrogenic differentiation. Osteogenic medium consists of IMDM supplemented with 0.1 μM dexamethasone, 10 mM β-glycerolphosphate and 0.2 mM ascorbic acid. Adipogenic medium consists of IMDM supplemented with 0.5 mM 3-isobutyl-1-methylxanthine, 1μM hydrocortisone, 0.1 mM indomethacin and 10% rabbit serum. Chondrogenic medium consists of DMEM supplemented with 6 μg/mL insulin–transferrin–selenium premix, 0.1 mM ascorbic acid 2-phosphate, 10 mM sodium pyruvate, 10 ng/mL transforming growth factor-β1 and 100 nM dexamethasone.

### The establishment of IUA model

Rabbit IUA models were constructed according to Liu et al. [22]. Rabbits (*n* = 9) were randomly subdivided into three groups, including control group (*n* = 3, rabbits without any treatment), 14 days after surgery group (*n* = 3, rabbits underwent IUA modeling surgery, killed on 14th day), and 28 days after surgery group (*n* = 3, rabbits underwent IUA modeling surgery, killed on 28th day).

**Fig. 1:**
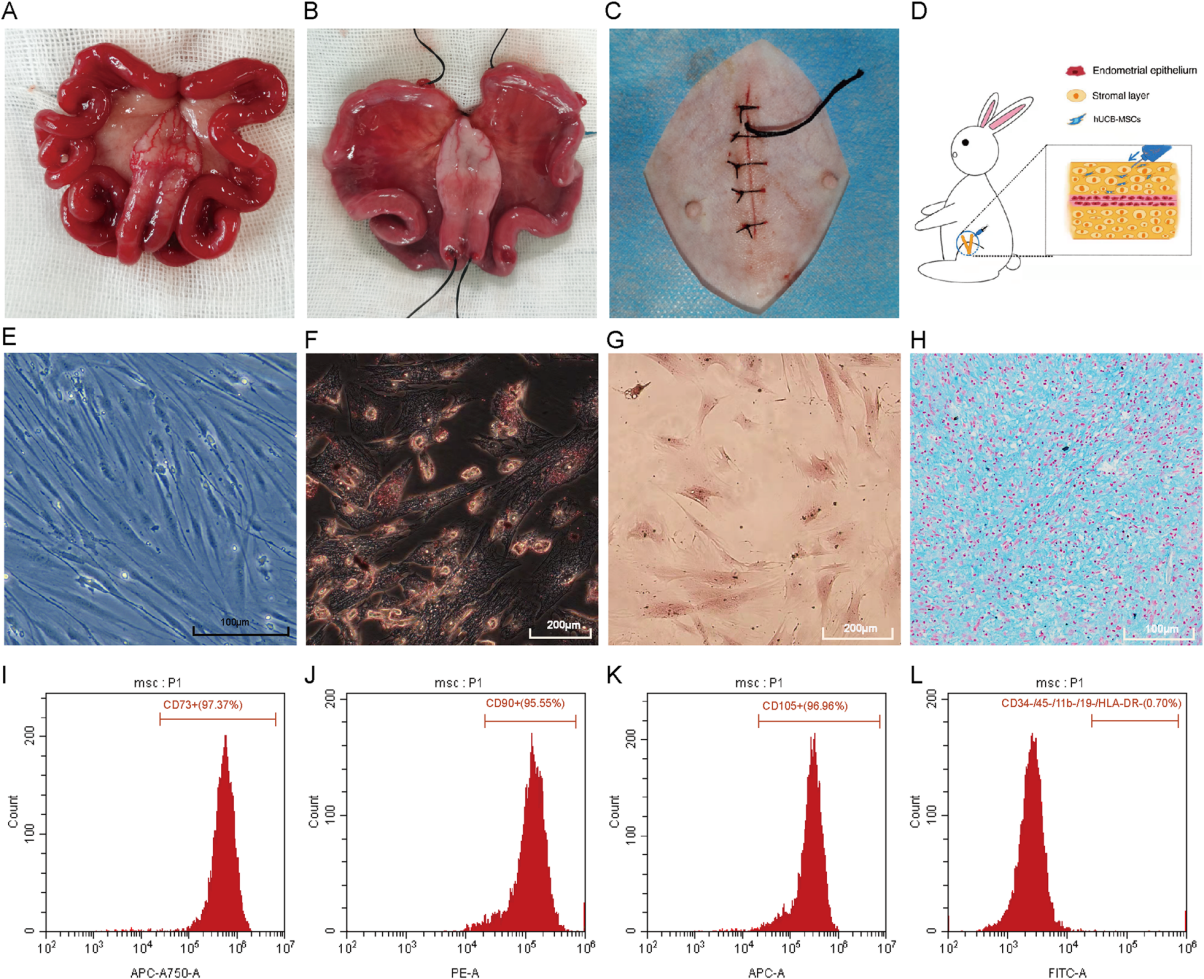
Establishment of IUA model and characterization of hUCB-MSCs. **A** Normal uterus after laparotomy without treatment. **B** Uterus after IUA surgery. An LPS surgical suture placed in the uterine cavity after curettage. **C** The abdomen after suture. **D** Schematic diagram of hUCB-MSCs injected into rabbit uterus wall. **E** Representative image of hUCB-MSCs. Scale bar: 100 μm. **F**, **G** Representative image of hUCB-MSCs adipogenic (**F**) and osteogenic (**G**) differentiation. Scale bar: 200 μm. **H** Representative image of hUCB-MSCs chondrogenic differentiation. Scale bar: 100 μm. **I**–**L** Surface antigens of hUCB-MSCs detected by flow cytometry assay. Cells were positive for CD73, CD90 and CD105, but negative for CD34, CD45, CD11b, CD19 and HLA-DR

All rabbits in model group were anesthetized with urethane (1.5 g/kg) through ear venous. They were then placed in a supine position, and the lower abdomen was shaved and sterilized with 70% ethanol on the operating table. A vertical incision (2.5–3 cm) was performed, and the bilateral uterine horns were exposed (Fig. 1A). A 0.5-cm longitudinal incision was scissored in the uterus to scrape the inner endometrium with a small curette until feeling rough. After curettage, an LPS surgical suture was placed in the uterine cavity (Fig. 1B). The uterine cavity and peritoneal cavity were thoroughly rinsed with physiological saline, and then the abdomen was sutured (Fig. 1C). The LPS surgical suture was removed at 48 h after surgery. On 14th and 28th day after surgery, three rabbits were killed for the collection of uterine tissue in model group, respectively.

### Treatment for IUA model

The rabbits (*n* = 6) were randomly assigned to two groups, including estrogen treatment group (*n* = 3, rabbits underwent surgery of intrauterine adhesions, estradiol benzoate (0.5 mg/kg) was administered intramuscularly every 4 days) and hUCB-MSCs treatment group (*n* = 3, rabbits underwent surgery of intrauterine adhesions, 1 weeks after surgery, a relaparotomy was performed and the rabbits were injected with 1 × 10^6^ hUCB-MSCs in each of the uterine wall, Fig. 1D). After 28 days of treatments, all rabbits were killed for the collection of uterine tissue.

### Histological analysis

The endometrial gland number and fibrosis rate were examined via hematoxylin–eosin (HE) and Masson staining, respectively. The excised uteri were fixed in 4% paraformaldehyde, then embedded in paraffin, sliced into 5-mm-thick sections, and routinely stained with HE and Masson stains according to standard protocols. Sections were examined under an inverted microscope (Leica, German, DMIL-PH1). Four high-power fields (HPF) were selected on each HE-stained slice to count the number of glands. Four high-power fields (HPF) were selected on each Masson-stained slice, and the fibrosis rate was calculated using ImageJ software (National Institutes of Health) as follows: the area of endometrial stromal fibrosis/the area of endometrial stroma and glands (excluding the uterine cavity).

### Immunohistochemistry

In order to evaluate expressions of endometrial receptivity-related estrogen receptor (ER), angiogenesis related Ki-67, and inflammation-related transforming growth factor-β1 (TGF-β1), immunohistochemistry was used. The transverse paraffined uterine sections were deparaffinized, rehydrated, and then incubated in 5% bovine serum albumin (Beyotime) for 30 min at 37 °C to block the nonspecific antibody. Sections of the uterus were deparaffinized and gradually dehydrated. Slides were incubated with rabbit anti-Ki-67 (1:200 dilution), anti-ER (1:200 dilution) and anti-TGF-β1 (1:200 dilution) monoclonal antibody at 4 °C overnight. And then the sections were incubated with goat anti-rabbit secondary antibodies for 60 min at room temperature, and the reaction was stopped with 3,3-diaminobenzidine. ImageJ software (National Institutes of Health) was used to evaluate the expression of Ki-67, ER and TGF-β1-positive cells, respectively, at a magnification of 400×. Five fields were randomly selected from each slide to determine the mean optical density (MOD).

### Single-cell RNA sequencing (scRNA-seq)

A rabbit IUA sample with hUCB-MSCs transplantation was utilized to single-cell RNA sequencing and bioinformatics analysis. The dually edited Mel-RM (Mel-RM.DE) cells were serum-starved for 96 h, and the endometrial quiescent cells were sorted, washed twice and re-suspended in cold PBS (calcium and magnesium free) with 0.04% FBS. Cell number and viability were determined using hemocytometer and trypan blue staining, and 1 × 10^5^ cells were subjected to 10 × Genomics sequencing according to the manufacturer’s protocol by Shanghai OE Biotech Co., Ltd. (Shanghai, China). Briefly, viable endometrial cells isolated from dually edited Mel-RM cells after serum starvation were analyzed using the 10 × Genomics Chromium Droplet platform with unique transcript counting through barcoding with unique molecular identifiers (UMIs). Cell Ranger 3.1.0 and Seurat 3.1.1 were used to analyze the sequencing results.

Considering that the rabbit IUA sample was treated with hUCB-MSCs, we mapped the sequenced reads to the human and rabbit reference genomes, respectively, obtaining 4363 human-derived cells and 10,599 rabbit-derived cells. After quality filtering to remove cells expressing high mitochondrial gene signatures and excluding doublets, 4097 human-derived cells and 8792 rabbit-derived cells were retained for further analysis. Upon gene expression normalization for read depth, cells were subjected to t-distributed stochastic neighbor embedding (t-SNE) and several unsupervised cell clusters were obtained and visualized using Loupe Browser. The cluster-specific markers were identified by detecting the differentially expressed genes between the given cluster and the other clusters.

Pseudotime trajectories were constructed with Monocle (version 2.6.4) [23]. Briefly, we first selected a set of ordering genes which showed differential expression between clusters. Then, monocle uses reversed graph embedding, a machine learning technique to learn a parsimonious principal graph, and reduces the given high-dimensional expression profiles to a low-dimensional space. Single cells are projected onto this space and ordered into a trajectory with branch points. As called in Monocle, cells in the same segment of the trajectory have the same ‘state.’

### RNA expression profiling by RNA sequencing

The rabbit IUA samples with and without treated with hUCB-MSCs were used for transcriptome sequencing (3 vs 3). Firstly, total RNAs were extracted by Trizol method, RNA purity was detected by spectrophotometer, and RNA integrity was analyzed by agarose gel electrophoresis and Agilent 2100 BioAnalyzer. The Library was constructed using Illumina’s NEBNext® UltraTM RNA Library Prep Kit. Then, Illumina platform was used for library sequencing and 150 bp paired terminal reading was generated to obtain the sequence information of the fragment to be measured. After quality control and sequence alignment based on reference genome, DESeq2 software [24] was used to analyze the differentially expressed genes (DEGs) between the two groups. Finally, the DEGs were used for gene enrichment analysis based on gene ontology (GO) and Kyoto Encyclopedia of Genes and Genomes (KEGG).

### Western blot

Proteins were extracted from endometrium samples with RIPA buffer containing proteinase inhibitors. The protein concentrations were quantified using BCA Protein Assay Kit (Beyotime, Shanghai, China) and separated in SDS-PAGE gel (Solarbio, Beijing), and then they were transferred onto the PVDF membrane. The membranes were incubated with anti-NF-κB-p65 and anti-GADPH for overnight at 4 °C, followed by secondary antibodies for 1 h at room temperature. The blots were visualized using ECL chemiluminescence kit (enhanced), and the band intensity was quantified with ImageJ software.

### qRT-PCR

Total RNA was extracted from excised endometrium using trizol reagent (Invitrogen, USA). cDNA was generated by reverse transcription of per RNA sample using PrimeScript RT reagent kits (Takara, Japan) following the manufacturer’s guidelines. The PCR reaction system was performed with specific primers and SYBR Premix Ex Taq II (Takara, Japan), with a final volume of 20 μL. The PCR cycling conditions were as follows: pre-denaturation at 95 °C for 30 s, denaturation at 95 °C for 5 s, annealing at 60 °C for 30 s, 40 cycles, and elongation at 60 °C for 30 s. Analyses of relative gene expressions were performed using 2^−ΔΔCT^ methods. GAPDH was used as an internal control. Primer sequences are summarized in Table 1.

**Table 1.** Primers of specific genes used in qRT-PCR analyses

Gene	Sequence (5′–3′)
NFKB1	
Forward	CCTGAGTCTTTTGGACCGCT
Reverse	GCAGGCTATTGCTCAACACG
GAPDH	
Forward	CTTTGGTATCGTGGAAGGA
Reverse	AGGGATGATGTTCTGGAGAG

### Statistical analysis

Statistical analysis was performed with SPSS 20.0 software. Numerical data were indicated as means standard deviation. For nonparametric statistics, data were analyzed using the Mann–Whitney U test and presented as populations with median values indicated by bars. For parametric statistics, data were analyzed using unpaired Student’s t test. Data were presented as a mean value with 95% confidence interval (CI). *P* values < 0.05 were considered to be statistically significant.

## Results

### Identification and characterization of hUCB-MSCs

We observed that hUCB-MSCs were adherent, with a fibroblast-like shape under optical microscope (Fig. 1E). Lipid droplets can be observed by Oil Red O staining (Fig. 1F); calcified extracellular matrix was detected by Alizarin Red S staining, (Fig. 1G); blue-stained acid proteoglycan was observed by Alcian Blue staining (Fig. 1H), demonstrating that hUCB-MSCs could differentiate into adipocytes, osteoblasts and chondroblasts. The results of flow cytometry showed that the cells were positive for CD73, CD90 and CD105, but negative for CD34, CD45, CD11b, CD19 and HLA-DR (Fig. 1I–L), indicating that these cells were MSCs, not hematopoietic stem cells or macrophages. These findings are concordant with the characteristics of mesenchymal stem cells.

### The gland number and fibrosis rate of endometrium after hUCB-MSCs transplantation

**Fig. 2:**
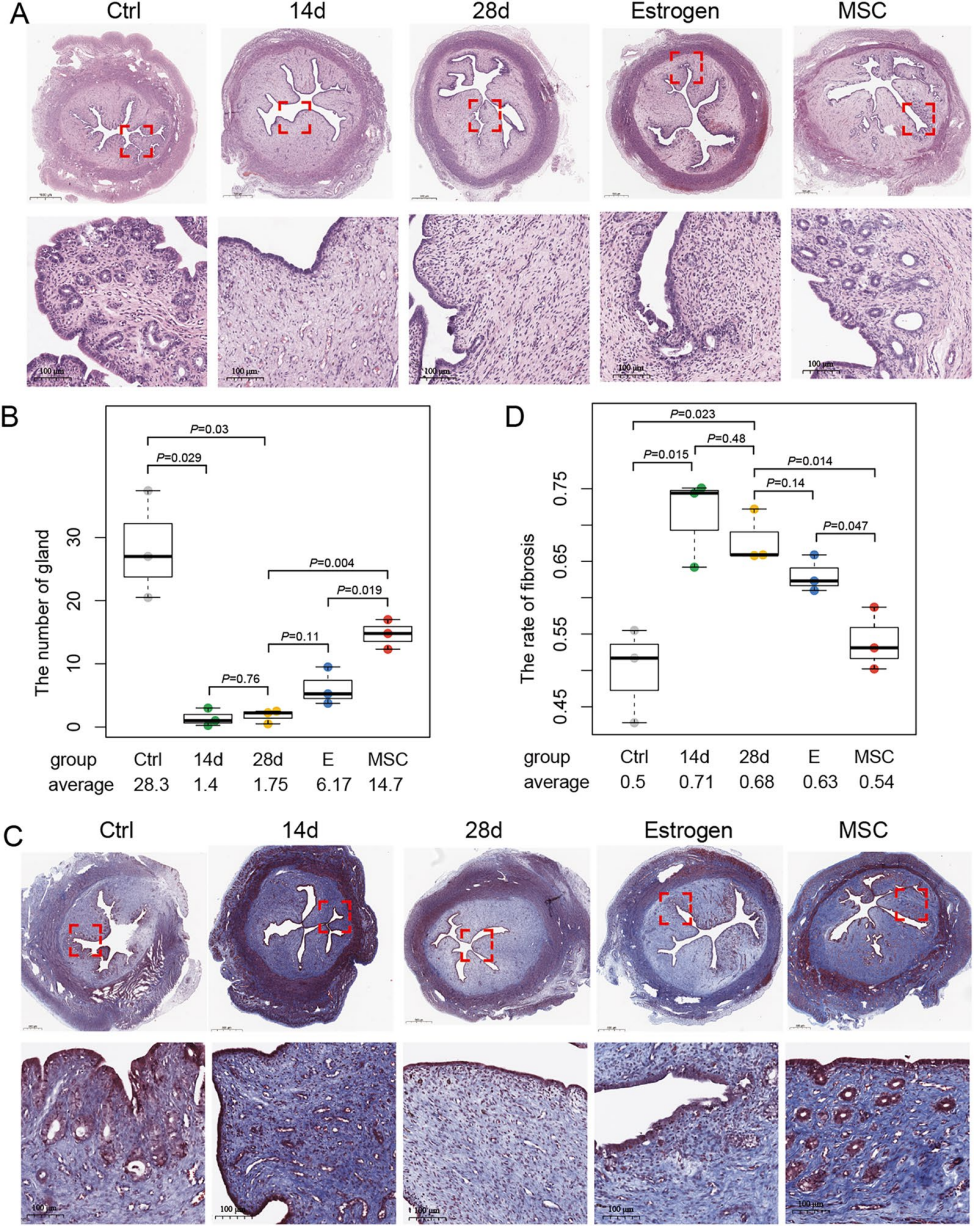
Histological analysis of uterine transverse section. **A** Micrographs representing HE-stained endometrial tissue from 5 groups, i.e., rabbits without surgery (control), 14/28th day after surgery, and treated with estrogen/hUCB-MSCs. Original magnification at ×400. **B** Endometrial gland number in 5 groups. **C** Micrographs representing Masson-stained endometrial tissue from 5 groups. Original magnification at ×400. **D** Endometrial fibrosis in 5 groups. Each group has three independent experiments

We found that normal endometrial surface was covered with simple high columnar epithelial cells, and endometrial glands were primarily located in submucosa and basal layer. On 14/28th day after surgery, the endometrium showed flat low columnar epithelial cells (Fig. 2A). Compared with control group, the gland number markedly reduced, and the fibrosis rate was obviously elevated in 14/28th day after surgery (*P* < 0.05, Fig. 2A–D). The situation did not improve on 28th day after surgery compared with 14th day, which indicated that the rabbit IUA models were successfully established.

After the hUCB-MSCs transplantation, the gland number statistically increased and the fibrosis rate decreased compared with 28th day after surgery (*P* < 0.05, Fig. 2A–D). Though the tendency of increased gland number and decreased fibrosis rate can be observed, there was still no significance on the two indexes between the estrogen treatment group and 28th day after surgery (*P* > 0.05, Fig. 2A–D). Compared with estrogen treatment group, the two indexes in hUCB-MSCs transplantation group were closer to control group, which suggested that hUCB-MSCs could be superior to estrogen in IUA treatment.

### Expression of ER, Ki-67 and TGF-β1 of endometrium after hUCB-MSCs transplantation

**Fig. 3:**
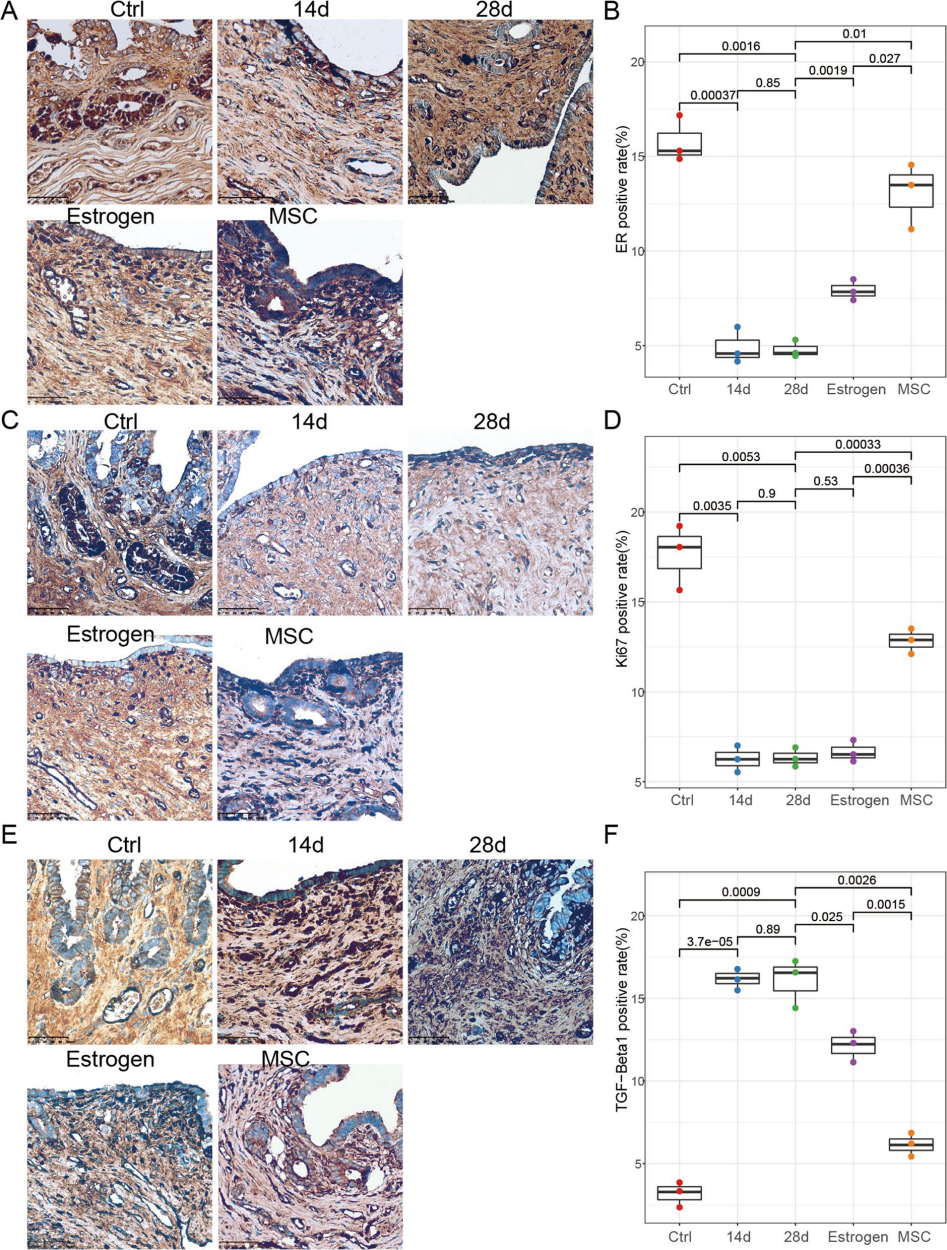
Immunohistochemistry of ER, Ki-67 and TGF-β1 (**A**, **C**, **E**). Micrographs of ER (**A**), Ki-67 (**C**) and TGF-β1 (**E**) expression from 5 groups, i.e., rabbits without surgery (control), 14/28th day after surgery, and treated with estrogen/hUCB-MSCs. (**B**, **D**, **F**). The expression of ER (**B**), Ki-67 (**D**) and TGF-β1 (**F**) in endometrium in 5 groups. All original magnification at ×400. Each group has three independent experiments

To further explore the hUCB-MSCs contributor on IUA endometrium, we detected the ER, Ki-67 and TGF-β1 expression in endometrial issues (Fig. 3A, C, E), which reflect the hormone level, the ability of angiogenesis and the degree of fibrosis of uterine tissue, respectively. After the hUCB-MSCs transplantation, there is an obvious increase in ER and Ki-67 expression and obvious reduction in TGF-β1 expression compared to 28th day after surgery (*P* < 0.05, Fig. 3B, D, F). Notably, compared with 28th day after surgery, the increase in ER expression and reduction in TGF-β1 expression were observed in estrogen treatment group (*P* < 0.05, Fig. 3B, F), but no significance in Ki-67 expression between the two groups (*P* > 0.05, Fig. 3D). Furthermore, compared with estrogen treatment group, the expression of the three protein in hUCB-MSCs transplantation group was more similar to control group. These pieces of evidence proved that hUCB-MSCs have advantages over estrogen for IUA treatment.

### ScRNA-seq revealed hUCB-MSCs trans-differentiate into endometrial cells

**Fig. 4:**
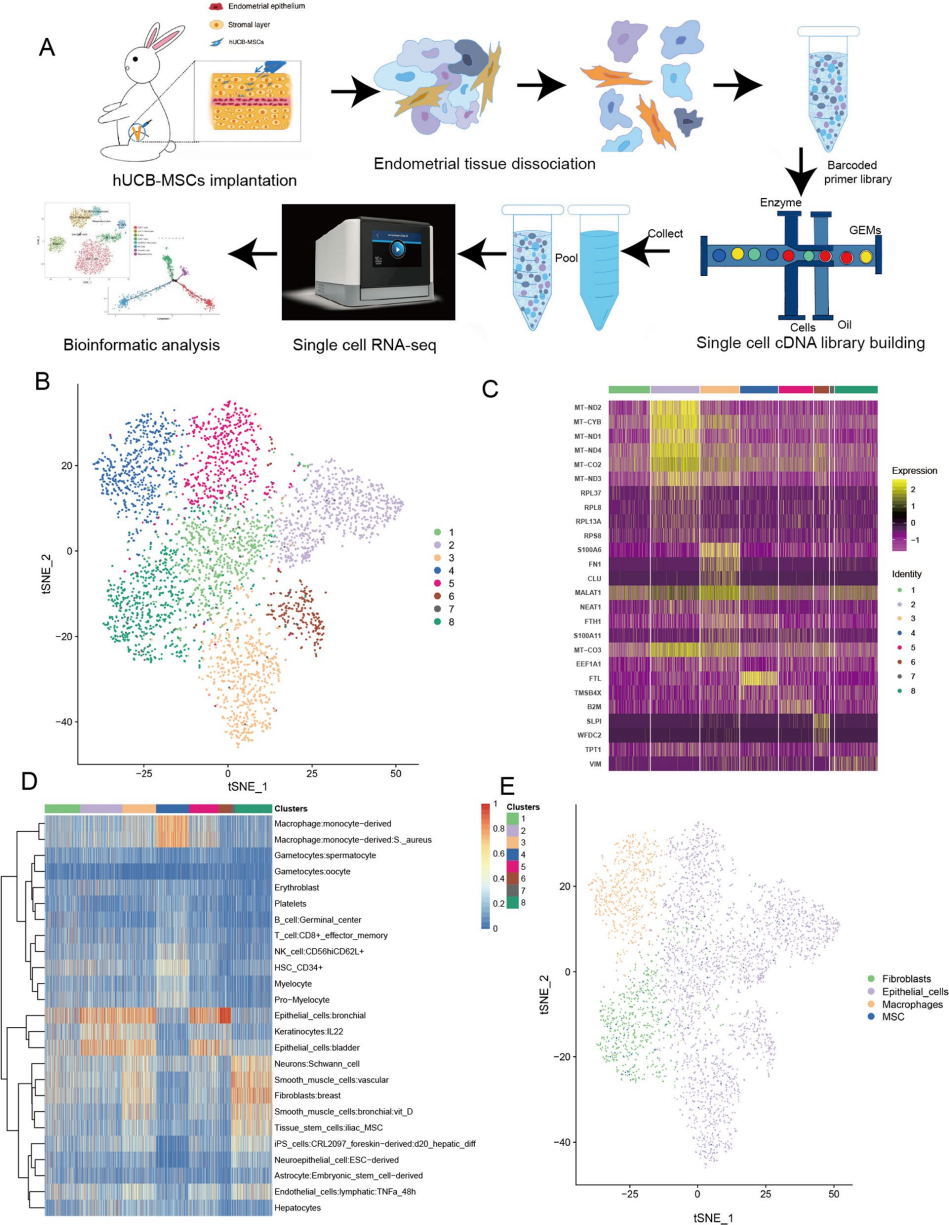
Identification of endometrium populations with single-cell transcriptomic analysis. **A** The workflow shows the collection and processing of obtained endometrium sample for scRNA-seq. **B** t-distributed stochastic neighbor (t-SNE) visualization of all human-derived cells displayed with different colors for clusters. **C** A heatmap revealed the expression levels of the indicated genes for human-derived cells. **D** The heatmap represents the corresponding relationship of 8 cell clusters and human-derived cell types; red (or blue) color indicates a high (or low) proportion. **E** t-SNE visualization of four main human-derived cell types after re-annotation

To investigate the cellular architecture and cell differentiation trajectory of IUA from single cell level, a rabbit IUA sample with hUCB-MSCs transplantation was utilized to scRNA-seq and bioinformatics analysis (Fig. 4A). After quality control, 4097 human-derived cells were used to perform t-SNE analysis and 8 cell clusters were obtained (Fig. 4B). By analyzing the differential expressed genes across the clusters and combining with classical markers of human cell types from a previous study [25], the relationship of the clusters and cell types was displayed (Fig. 4C, D). After purification and re-annotation manually, four main cell types including MSCs (68 cells, 1.66%), fibroblasts (664 cells, 16.21%), epithelial cells (2770 cells, 67.61%) and macrophages (595 cells, 14.52%) were revealed (Fig. 4E). The MSCs annotated were the residual transplanted hUCB-MSCs, which did not take long enough to transform into any other cell types, while the remaining three cell types (fibroblasts, epithelial cells and macrophages) could be the result of endometrial reconstruction after hUCB-MSCs transplantation for IUA treatment.

**Fig. 5:**
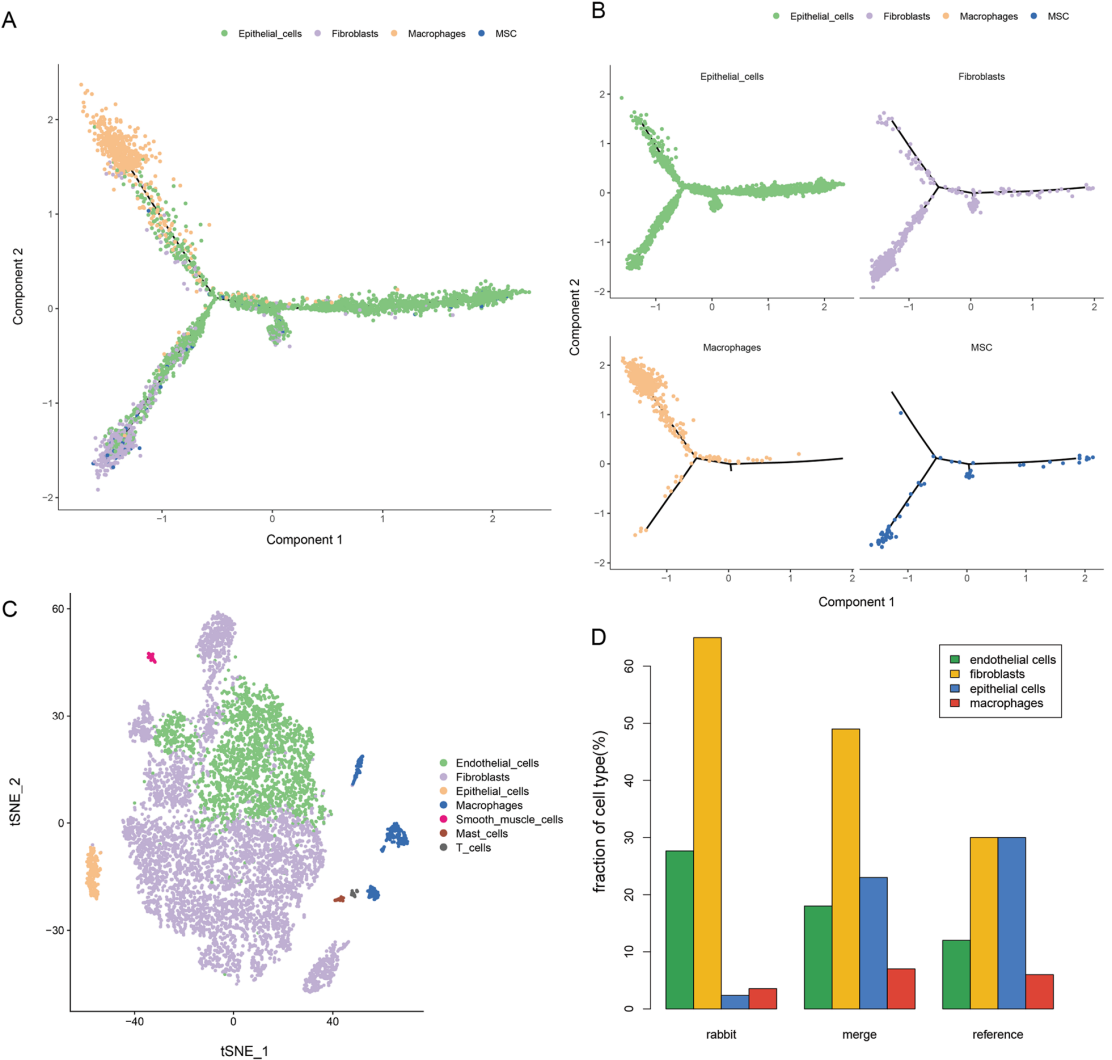
Cell differentiation trajectory of hUCB-MSCs and the cell type proportion of rabbit-derived cells. **A** The trajectory with 3 branches identified by pseudotime analysis. **B** The specific cell distribution of four cell types in differentiation trajectory. **C** Four mainly cell types and three rare cell types identified in rabbit-derived cells. **D** The proportion of four mainly cell types in IUA rabbit-derived cells, merged (human and rabbit) endometrial cells and normal human endometrial cells from a reference literature

Since MSCs displayed stem cell-like signatures and have potential to differentiate into other cell types, we speculated that pseudotime series analysis based on the four cell types may capture the main differentiation processes of MSCs during treatment of IUA using hUCB-MSCs. A cell trajectory was reconstructed by Monocle, which mainly contained three branches (Fig. 5A). Notably, the trajectory’s root was MSCs, which trans-differentiate into epithelial cells throughout the whole process. And some MSCs trans-differentiate into fibroblasts firstly, while some followed to trans-differentiate into macrophages (Fig. 5B). The results suggested that hUCB-MSCs can trans-differentiate into endometrial tissues, which will benefit for the mechanism understanding and clinical treatment of MSCs transplantation in uterine inflammatory injury.

Similarly, 8792 rabbit-derived cells were annotated to four main cell types: endothelial cells (2431 cells, 27.65%), fibroblasts (5721 cells, 65.07%), epithelial cells (208 cells, 2.37%) and macrophages (312 cells, 3.55%) and three rare cell types (Fig. 5C). Integrated human with rabbit-derived cells, we observed that the proportion of endothelial cells (2431 cells, 18.7%) and fibroblasts (6385 cells, 49.54%) was elevated, while the proportion of epithelial cells (2978, 23.1%) and macrophages (907 cells, 7.04%) was decreased (Fig. 5D). By analyzing the percentage of normal uterine tissue cells in a previous study [25], we found that the distribution of merged cell types was more similar to that of normal endometrium compared with that of rabbit-derived cell types (Fig. 5D). This indicates that MSCs can differentiate endometrial tissue cells and restore the damaged endometrial environment to normal, which reflects the value of mesenchymal stem cell transplantation in the treatment of IUA. With the prolongation of action time after MSC transplantation and the initiation of body self-repair, the proportion of cells in the damaged endometrial tissue may tend to be more and more normal.

### RNA Sequencing revealed hUCB-MSCs regulate Th17/Treg balance and NF-κB signaling in treating IUA

**Fig. 6:**
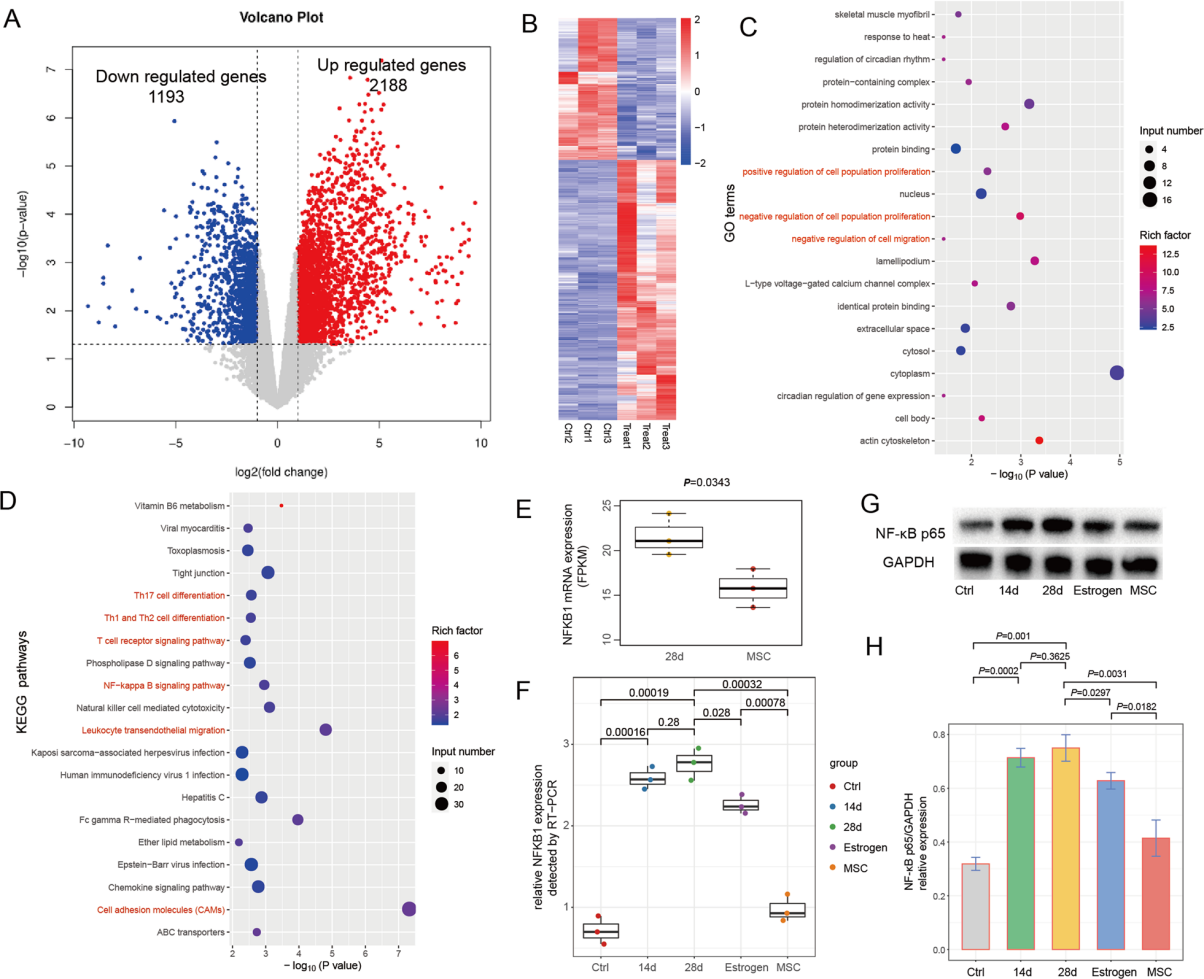
RNA sequencing and bioinformatics analysis reveal biological pathways involved in IUA treated with hUCB-MSCs. **A** The volcano plot of differential expressed genes in IUA before and after hUCB-MSCs transplantation. Red (or blue) color indicates up-regulated (or down-regulated) genes. **B** The heatmap of representative differential expressed genes. **C** The enriched GO terms in GO enrichment analysis. **D** The enriched KEGG pathways in pathway enrichment analysis. **E** The NFKB1 mRNA expression (FPKM) measured by RNA sequencing in two groups, each group has three samples. **F** The NFKB1 mRNA expression level detected by qRT-PCR assay in 5 groups, each group has three samples. **G** The representative WB image of NF-κB p65 protein expression. **H** The NF-κB p65 protein expression level measured by WB in 5 groups, i.e., rabbits without surgery (control), 14/28th day after surgery, and treated with estrogen/hUCB-MSCs

To clarify the biological role of treatment with hUCB-MSCs in IUA, RNA sequencing of 6 samples (3 for 28th day after surgery and 3 for hUCB-MSCs treatment) was performed to observe the molecular expression changes in IUA samples before and after hUCB-MSCs transplantation. We found that hUCB-MSCs treatment caused 2188 genes to be up-regulated and 1193 genes to be down-regulated (Fig. 6A, B). GO and KEGG pathway enrichment analysis was performed based on these differential expressed genes. GO result showed that treatment-associated genes were mainly enriched in cell proliferation and cell migration (Fig. 6C). KEGG analysis depicted that hUCB-MSCs treatment may participate in the pathways related to immune cells (Th1, Th2 and Th17) differentiation, T cell receptor signaling and NF-κB signaling pathway (Fig. 6D).

NF-κB signaling has been reported to be possible involved in MSCs transplantation to treat IUA [11], thus becoming to our focus. NFKB1 mRNA expression was significantly decreased after hUCB-MSCs treatment in RNA-Seq experiment (Fig. 6E), which was validated by qRT-PCR assay (Fig. 6F). WB assay also confirmed that NF-κB-p65 protein level indeed reduced compared with pre-treatment (28th day after surgery) (Fig. 6G, H), indicating that NF-κB signaling may play a crucial role in hUCB-MSCs regulating inflammatory microenvironment of endometrium. According to previous studies [25, 26], MSCs can regulate the polarization of naive T cells into anti-inflammatory regulatory T cells (Treg) and reduce the ratio of Th17/Treg cells to exert immunomodulatory function, which can mediate through the activation of NF-κB signaling pathway. Thus, we believed that hUCB-MSCs can regulate Th17/Treg balance and NF-κB signaling in treating IUA, which promoting the mechanism understanding and potential clinical application for treating IUA with hUCB-MSCs transplantation.

## Discussion

The components of endometrial tissue include epithelial cells, stromal cells, vascular smooth cells and vascular endothelial cells. The healthy endometrium with regenerative capacity can divide into two zones: the upper functional layer and lower basal layer. During a menstrual cycle, the functional layer shed, while the permanent basal layer can regenerate into new functional layer every menses as well as postpartum [27–29]. It has become evident that endometrial stem cells are responsible for endometrial regeneration [30], while their number of endometrial stem cells decreases and their function was weaken in IUAs due to the damaged basal layers [1]. Exogenous MSCs transplantation may compensate for this endogenous reduction to exert the role of regeneration.

MSCs have effects on tissue repair by homing to the injured site, secreting chemokines, modulating the immune function, differentiating into other types of cells and potentially having antimicrobial ability. They can suppress inflammation by secreting immunomodulatory factors, such as, IL-6, IL-8, MCP-1, CCL5 and TLR-4, thereby decreasing proliferation and activation of CD4 + T cells [31, 32]. Clinical trials have demonstrated the initial safety and efficacy of mesenchymal stem cells derived from bone marrow, umbilical cord, menstrual blood and adipose tissue in restoring menstruation, fertility outcomes and endometrial regeneration. Studies have showed transplantation with the MSCs promoted endometrial regeneration and collagen resurgery and enhanced the expression of estrogen receptor of IUA model [32]. Animal experiments have corroborated that MSCs transplanted into uterine cavity of mice can exert immunomodulatory effects by secreting anti-inflammatory cytokines IL-6 [33]. Current knowledge on IUA pathogenesis was mostly derived from tissue studies without considering the multicellular structures and their orchestration; thus it is quite essential for in-depth mechanistic investigations, such as trans-differentiation, immunoregulation and signaling pathway.

Previous studies showed MSC-induced functional improvement in injured tissues by paracrine effect rather than direct differentiation [34, 35]. However, our study revealed the potential of differentiation for MSCs in tissue repair. The annotation analysis of human-derived cell types from scRNA-seq showed that hUCB-MSCs may differentiate fibroblasts, epithelial cells and macrophages (Fig. 4E), suggesting the potential of endometrial repair and regeneration after hUCB-MSCs transplantation in IUA treatment. (1) Previous studies demonstrated that MSCs transplantation can affect the composition and morphology of endometrial epithelial cells, and promote the regeneration of endometrial epithelial cells [36, 37]; (2) MSCs were considered as one of the sources for fibroblasts and even immature fibroblasts, which could regenerate during the repair of inflammatory damage [38]; (3) MSCs were reported to promote macrophage polarization inhibit inflammatory progression, and increased anti-inflammatory responses in vivo and in vitro [39]. These pieces of evidence also provide clues of the differentiation possibility into the three cell types during IUA treatment after MSCs transplantation. In our study, the pseudotime series analysis also showed MSCs can trans-differentiate into epithelial cells, fibroblasts and macrophages throughout the differentiation process or in different periods (Fig. 5A, B). The results suggested that scRNA-seq could reveal the cellular architecture and evolution of IUA from single cell level.

Excitingly, compared with rabbit-derived cell types, we found that the distribution of merged cell types was more similar to that of normal endometrium (Fig. 5D).The merged cell types represent IUA endometrium after hUCB-MSCs transplantation, while the rabbit-derived cell types roughly represent IUA endometrium without treatment. This indicates that MSCs can differentiate endometrial tissue cells and restore the damaged endometrial environment to normal. Of course, the proportion of merged cell types was still slightly different with normal endometrium. This could be due to the influence of distinct sample sources and processing methods, as well as the diversity of cell type annotations. On the other hand, we speculated that the proportion of cells in the damaged endometrial tissue may tend to be completely normal as MSCs continue to function as well as body self-repair.

MSCs can regulate the polarization of naive T cells into anti-inflammatory regulatory T cells (Treg) and reduce the ratio of Th17/Treg cells to exert immunomodulatory function [25, 40, 41]. The immunomodulatory effect of MSCs is proved to mainly communicate with T cell through IL-6-mediated paracrine way [42]. The interaction between lymphocytes and MSCs also depends on the induction of IL-6 expression by MSCs [43]. IL-6 has been demonstrated to regulate Th17/Treg balance [44], and inhibiting its expression could significantly impair the immunomodulatory function of MSCs [45]. On the other hand, MSC transplantation can mediate T cell response through the activation of NF-κB signaling pathway [26], which may rely on LAT to exhibit T cell regulatory function [46, 47]. The inhibition of LAT reduced CTLA-4 and CD25 expression in Treg cells and impairs their immunosuppression capacity [48]. These pieces of evidence indicate the key role of cellular molecules and signaling pathways in inflammation and immune regulation after MSCs transplantation [49]. In our study, functional enrichment analysis based on differential expressed genes showed that MSCs transplantation may treat IUA through T cell differentiation and NF-kappa B signaling pathway. In order to determine the immunomodulation function, the supernatants of third-generation hUCB-MSCs were collected and Legend plex TM human Th cytokine assay (BioLegend) was used to analyze the following 13 factors: IL-2, IL-4, IL-5, IL-6, IL-9, IL-10, IL-13, IL-17A, IL-17F, IL-21, IL-22, IFN-α and TNF-γ. Experiments were repeated to ensure the accuracy of the data. Finally, CytoFLEX flow cytometer (Beckman Coulter GmbH, Krefeld, Germany) was used for analysis, and the data were analyzed by LEGENDplex V8.0 and Prism7.0 software (BioLegend), the concentration was pg/ml. The results showed that the concentrations of IL-5, IL-13, TNF-α and IFN-γ were lower, the concentrations of IL-2, IL-9, IL-10, IL-17A, IL-17F, IL-4, IL-21 and IL-22 were undetectable, but the secretion level of IL-6 (891.58 pg/mL) was very high (Additional file 1: Figure S1-2). The expression of IL-6-related genes (IL6R and IL6ST) and LAT detected by RNA sequencing were significantly elevated compared with pre-treatment (Additional file 1: Figure S3). Therefore, we speculate that except for NFKB1, IL-6 and LAT may also be the key factors in the action mechanism of MSCs transplantation in the treatment of IUA, which needs to be further investigation in future studies.

## Conclusion

Transplantation of hUCB-MSCs promoted structural reconstruction and regulated inflammatory environment in IUA endometrium through NF-κB signaling. We firstly demonstrated that hUCB-MSCs can differentiate into normal endometrial tissue in rabbit. It was proved that hUCB-MSCs may be a promising therapy for humans with IUA. Of course, more research is needed on the long-term safety profile and effectiveness for patients with IUA.

## Supplementary Information

**Additional file 1:** The concentration and expression analysis of cytokines in Discussion.

## Data Availability

The RNA-seq and scRNA-seq datasets can be available in GEO with accession number GSE205997. The original images and statistic tables were attached in Additional file 1.

## Abbreviations

hUCB-MSCs: Human umbilical cord blood-derived mesenchymal stem cells
ER: Estrogen receptor
H&E: Hematoxylin and eosin
WB: Western blot
IUA: Intrauterine adhesion
TGF: Transforming growth factor
IL: Interleukin
MCP: Monocyte chemoattractant protein
CCL: Chemokine ligand
TLR: Toll-like receptor
LAT: Linker for activation of T cells

## References

[1] Yu D, Wong YM, Cheong Y, Xia E, Li TC (2008). Asherman syndrome–one century later. Fertil Steril.

[2] Deans R, Abbott J (2010). Review of intrauterine adhesions. J Minim Invasive Gynecol.

[3] Conforti A, Alviggi C, Mollo A, De Placido G, Magos A (2013). The management of Asherman syndrome: a review of literature. Reprod Biol Endocrinol.

[4] Johary J, Xue M, Zhu X, Xu D, Velu PP (2014). Efficacy of estrogen therapy in patients with intrauterine adhesions: systematic review. J Minim Invasive Gynecol.

[5] Lin X, Zhang Y, Pan Y, He S, Dai Y, Zhu B, Wei C, Xin L, Xu W, Xiang C (2018). Endometrial stem cell-derived granulocyte-colony stimulating factor attenuates endometrial fibrosis via sonic hedgehog transcriptional activator Gli2. Biol Reprod.

[6] Chen L, Zhang H, Wang Q, Xie F, Gao S, Song Y, Dong J, Feng H, Xie K, Sui L (2017). Reproductive outcomes in patients with intrauterine adhesions following hysteroscopic adhesiolysis: experience from the largest women's Hospital in China. J Minim Invasive Gynecol.

[7] Strug M, Aghajanova L (2021). Making more womb: clinical perspectives supporting the development and utilization of mesenchymal stem cell therapy for endometrial regeneration and infertility. J Pers Med.

[8] Xia L, Meng Q, Xi J, Han Q, Cheng J, Shen J, Xia Y, Shi L (2019). The synergistic effect of electroacupuncture and bone mesenchymal stem cell transplantation on repairing thin endometrial injury in rats. Stem Cell Res Ther.

[9] Chi Y, He P, Lei L, Lan Y, Hu J, Meng Y, Hu L (2018). Transdermal estrogen gel and oral aspirin combination therapy improves fertility prognosis via the promotion of endometrial receptivity in moderate to severe intrauterine adhesion. Mol Med Rep.

[10] Zhu Y, Hu J, Yu T, Ren Y, Hu L (2016). High molecular weight hyaluronic acid inhibits fibrosis of endometrium. Med Sci Monit.

[11] Xue X, Chen Q, Zhao G, Zhao JY, Duan Z, Zheng PS (2015). The Overexpression of TGF-beta and CCN2 in intrauterine adhesions involves the NF-kappaB Signaling Pathway. PLoS ONE.

[12] Wang X, Ma N, Sun Q, Huang C, Liu Y, Luo X (2017). Elevated NF-kappaB signaling in Asherman syndrome patients and animal models. Oncotarget.

[13] Placek K, Schultze JL, Aschenbrenner AC (2019). Epigenetic reprogramming of immune cells in injury, repair, and resolution. J Clin Invest.

[14] Chen JM, Huang QY, Zhao YX, Chen WH, Lin S, Shi QY (2021). The latest developments in immunomodulation of mesenchymal stem cells in the treatment of intrauterine Adhesions. Both allogeneic and autologous. Front Immunol.

[15] Sagaradze GD, Basalova NA, Efimenko AY, Tkachuk VA (2020). Mesenchymal stromal cells as critical contributors to tissue regeneration. Front Cell Dev Biol.

[16] Hou X, Liu Y, Streuli I, Dallenbach P, Dubuisson J, Ansaldi Y, Pluchino N (2019). Endometrial regeneration in asherman's syndrome: clinical and translational evidence of stem cell therapies. Curr Stem Cell Res Ther.

[17] Lee OK, Kuo TK, Chen WM, Lee KD, Hsieh SL, Chen TH (2004). Isolation of multipotent mesenchymal stem cells from umbilical cord blood. Blood.

[18] Stiner R, Alexander M, Liu G, Liao W, Liu Y, Yu J, Pone EJ, Zhao W, Lakey JRT (2019). Transplantation of stem cells from umbilical cord blood as therapy for type I diabetes. Cell Tissue Res.

[19] Jeon HJ, Yoon KA, An ES, Kang TW, Sim YB, Ahn J, Choi EK, Lee S, Seo KW, Kim YB (2020). Therapeutic effects of human umbilical cord blood-derived mesenchymal stem cells combined with cartilage acellular matrix mediated via bone morphogenic protein 6 in a rabbit model of articular cruciate ligament transection. Stem Cell Rev Rep.

[20] Yang S, Wei Y, Sun R, Lu W, Lv H, Xiao X, Cao Y, Jin X, Zhao M (2020). Umbilical cord blood-derived mesenchymal stromal cells promote myeloid-derived suppressor cell proliferation by secreting HLA-G to reduce acute graft-versus-host disease after hematopoietic stem cell transplantation. Cytotherapy.

[21] Dominici M, Le Blanc K, Mueller I, Slaper-Cortenbach I, Marini F, Krause D, Deans R, Keating A, Prockop D, Horwitz E (2006). Minimal criteria for defining multipotent mesenchymal stromal cells. The International Society for Cellular Therapy position statement. Cytotherapy.

[22] Liu F, Zhu ZJ, Li P, He YL (2013). Creation of a female rabbit model for intrauterine adhesions using mechanical and infectious injury. J Surg Res.

[23] Qiu X, Mao Q, Tang Y, Wang L, Chawla R, Pliner HA, Trapnell C (2017). Reversed graph embedding resolves complex single-cell trajectories. Nat Methods.

[24] Love MI, Huber W, Anders S (2014). Moderated estimation of fold change and dispersion for RNA-seq data with DESeq2. Genome Biol.

[25] Chen QH, Wu F, Liu L, Chen HB, Zheng RQ, Wang HL, Yu LN (2020). Mesenchymal stem cells regulate the Th17/Treg cell balance partly through hepatocyte growth factor in vitro. Stem Cell Res Ther.

[26] Dorronsoro A, Ferrin I, Salcedo JM, Jakobsson E, Fernandez-Rueda J, Lang V, Sepulveda P, Fechter K, Pennington D, Trigueros C (2014). Human mesenchymal stromal cells modulate T-cell responses through TNF-alpha-mediated activation of NF-kappaB. Eur J Immunol.

[27] Alawadhi F, Du H, Cakmak H, Taylor HS (2014). Bone Marrow-Derived Stem Cell (BMDSC) transplantation improves fertility in a murine model of Asherman's syndrome. PLoS ONE.

[28] Santamaria X, Cabanillas S, Cervello I, Arbona C, Raga F, Ferro J, Palmero J, Remohi J, Pellicer A, Simon C (2016). Autologous cell therapy with CD133+ bone marrow-derived stem cells for refractory Asherman's syndrome and endometrial atrophy: a pilot cohort study. Hum Reprod.

[29] Cervello I, Gil-Sanchis C, Santamaria X, Cabanillas S, Diaz A, Faus A, Pellicer A, Simon C (2015). Human CD133(+) bone marrow-derived stem cells promote endometrial proliferation in a murine model of Asherman syndrome. Fertil Steril.

[30] Masuda H, Matsuzaki Y, Hiratsu E, Ono M, Nagashima T, Kajitani T, Arase T, Oda H, Uchida H, Asada H (2010). Stem cell-like properties of the endometrial side population: implication in endometrial regeneration. PLoS ONE.

[31] Cortes-Araya Y, Amilon K, Rink BE, Black G, Lisowski Z, Donadeu FX, Esteves CL (2018). Comparison of antibacterial and immunological properties of mesenchymal stem/stromal cells from equine bone marrow, endometrium, and adipose tissue. Stem Cells Dev.

[32] Queckborner S, Syk Lundberg E, Gemzell-Danielsson K, Davies LC (2020). Endometrial stromal cells exhibit a distinct phenotypic and immunomodulatory profile. Stem Cell Res Ther.

[33] Gan L, Duan H, Xu Q, Tang YQ, Li JJ, Sun FQ, Wang S (2017). Human amniotic mesenchymal stromal cell transplantation improves endometrial regeneration in rodent models of intrauterine adhesions. Cytotherapy.

[34] Wakabayashi K, Hamada C, Kanda R, Nakano T, Io H, Horikoshi S, Tomino Y (2014). Adipose-derived mesenchymal stem cells transplantation facilitate experimental peritoneal fibrosis repair by suppressing epithelial-mesenchymal transition. J Nephrol.

[35] Bandeira F, Goh TW, Setiawan M, Yam GH, Mehta JS (2020). Cellular therapy of corneal epithelial defect by adipose mesenchymal stem cell-derived epithelial progenitors. Stem Cell Res Ther.

[36] Lu X, Cui J, Cui L, Luo Q, Cao Q, Yuan W, Zhang H (2019). The effects of human umbilical cord-derived mesenchymal stem cell transplantation on endometrial receptivity are associated with Th1/Th2 balance change and uNK cell expression of uterine in autoimmune premature ovarian failure mice. Stem Cell Res Ther.

[37] Zhang L, Li Y, Guan CY, Tian S, Lv XD, Li JH, Ma X, Xia HF (2018). Therapeutic effect of human umbilical cord-derived mesenchymal stem cells on injured rat endometrium during its chronic phase. Stem Cell Res Ther.

[38] Soundararajan M, Kannan S (2018). Fibroblasts and mesenchymal stem cells: Two sides of the same coin?. J Cell Physiol.

[39] Xin L, Lin X, Zhou F, Li C, Wang X, Yu H, Pan Y, Fei H, Ma L, Zhang S (2020). A scaffold laden with mesenchymal stem cell-derived exosomes for promoting endometrium regeneration and fertility restoration through macrophage immunomodulation. Acta Biomater.

[40] Galipeau J, Sensebe L (2018). Mesenchymal stromal cells: clinical challenges and therapeutic opportunities. Cell Stem Cell.

[41] Weiss ARR, Dahlke MH (2019). Immunomodulation by Mesenchymal Stem Cells (MSCs): mechanisms of action of living, apoptotic, and dead MSCs. Front Immunol.

[42] Song N, Scholtemeijer M, Shah K (2020). Mesenchymal stem cell immunomodulation: mechanisms and therapeutic potential. Trends Pharmacol Sci.

[43] Xu G, Zhang Y, Zhang L, Ren G, Shi Y (2007). The role of IL-6 in inhibition of lymphocyte apoptosis by mesenchymal stem cells. Biochem Biophys Res Commun.

[44] Kimura A, Kishimoto T (2010). IL-6: regulator of Treg/Th17 balance. Eur J Immunol.

[45] Dorronsoro A, Lang V, Ferrin I, Fernandez-Rueda J, Zabaleta L, Perez-Ruiz E, Sepulveda P, Trigueros C (2020). Intracellular role of IL-6 in mesenchymal stromal cell immunosuppression and proliferation. Sci Rep.

[46] Keller B, Zaidman I, Yousefi OS, Hershkovitz D, Stein J, Unger S, Schachtrup K, Sigvardsson M, Kuperman AA, Shaag A (2016). Early onset combined immunodeficiency and autoimmunity in patients with loss-of-function mutation in LAT. J Exp Med.

[47] Malissen B, Aguado E, Malissen M (2005). Role of the LAT adaptor in T-cell development and Th2 differentiation. Adv Immunol.

[48] Shen S, Chuck MI, Zhu M, Fuller DM, Yang CW, Zhang W (2010). The importance of LAT in the activation, homeostasis, and regulatory function of T cells. J Biol Chem.

[49] Thaker YR, Schneider H, Rudd CE (2015). TCR and CD28 activate the transcription factor NF-kappaB in T-cells via distinct adaptor signaling complexes. Immunol Lett.

