# Supplementary material for: Human umbilical cord blood-derived MSCs trans-differentiate into endometrial cells and regulate Th17/Treg balance through NF-κB signaling in rabbit intrauterine adhesions endometrium
Source: Stem Cell Res Ther. 2022 Jul 15;13:301. doi: 10.1186/s13287-022-02990-1 (PMC9284747; doi:10.1186/s13287-022-02990-1)
Supplement: Supplementary file 1 — Additional file 1: The concentration and expression analysis of cytokines in Discussion. [file 13287_2022_2990_MOESM1_ESM.docx]

**The Supplemental Figures of DOI: 10.1186/s13287-022-02990-1**

**Title:** **Human umbilical cord blood-derived MSCs trans-differentiate into endometrial cells and regulate Th17/Treg balance through NF-κB signaling in rabbit intrauterine adhesions endometrium**

**The concentration and expression analysis of cytokines in Discussion**


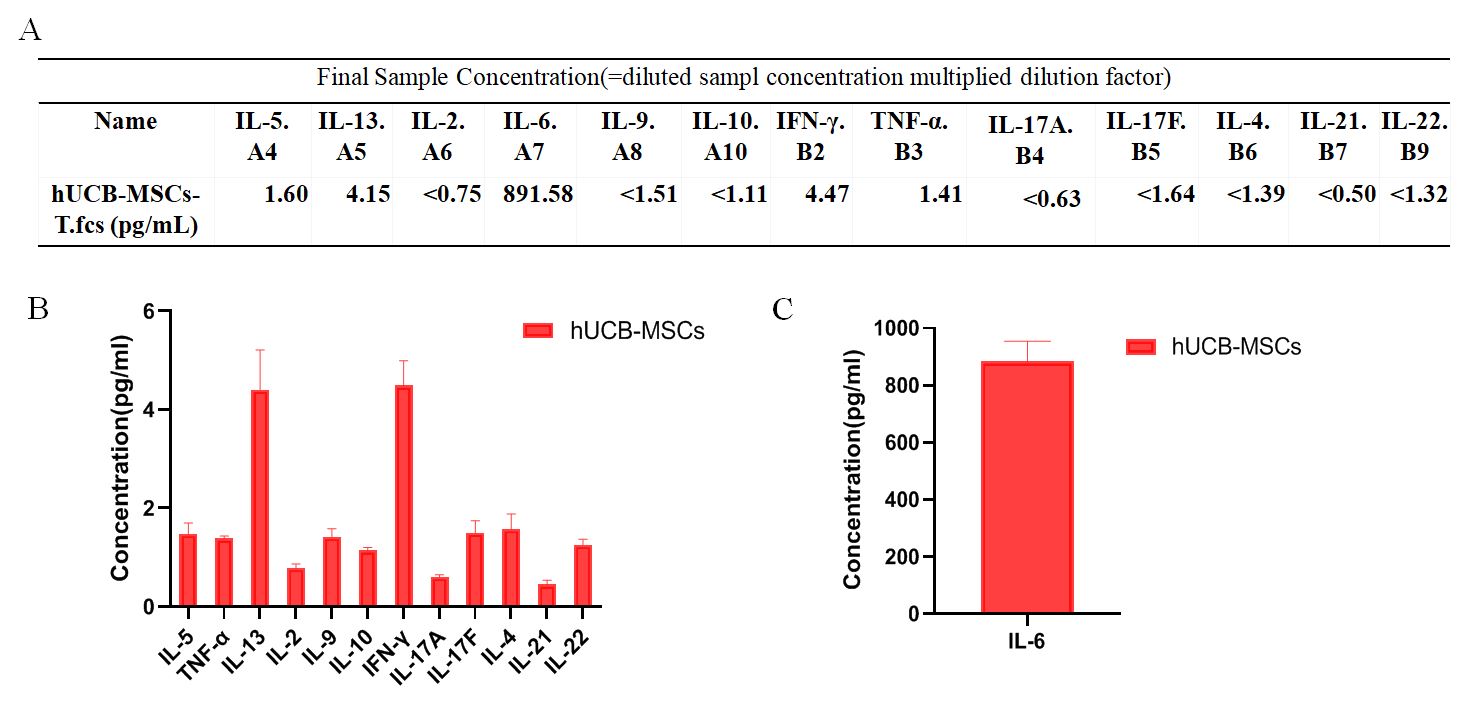


**Supplemental Figure 1. Cytokine concentrations of P3 hUCB-MSCs. (A)The table of all cytokine concentrations were detected in the media of P3 hUCB-MSCs. (B-C) The barplot of all cytokine concentrations were detected in the media of P3 hUCB-MSCs.**


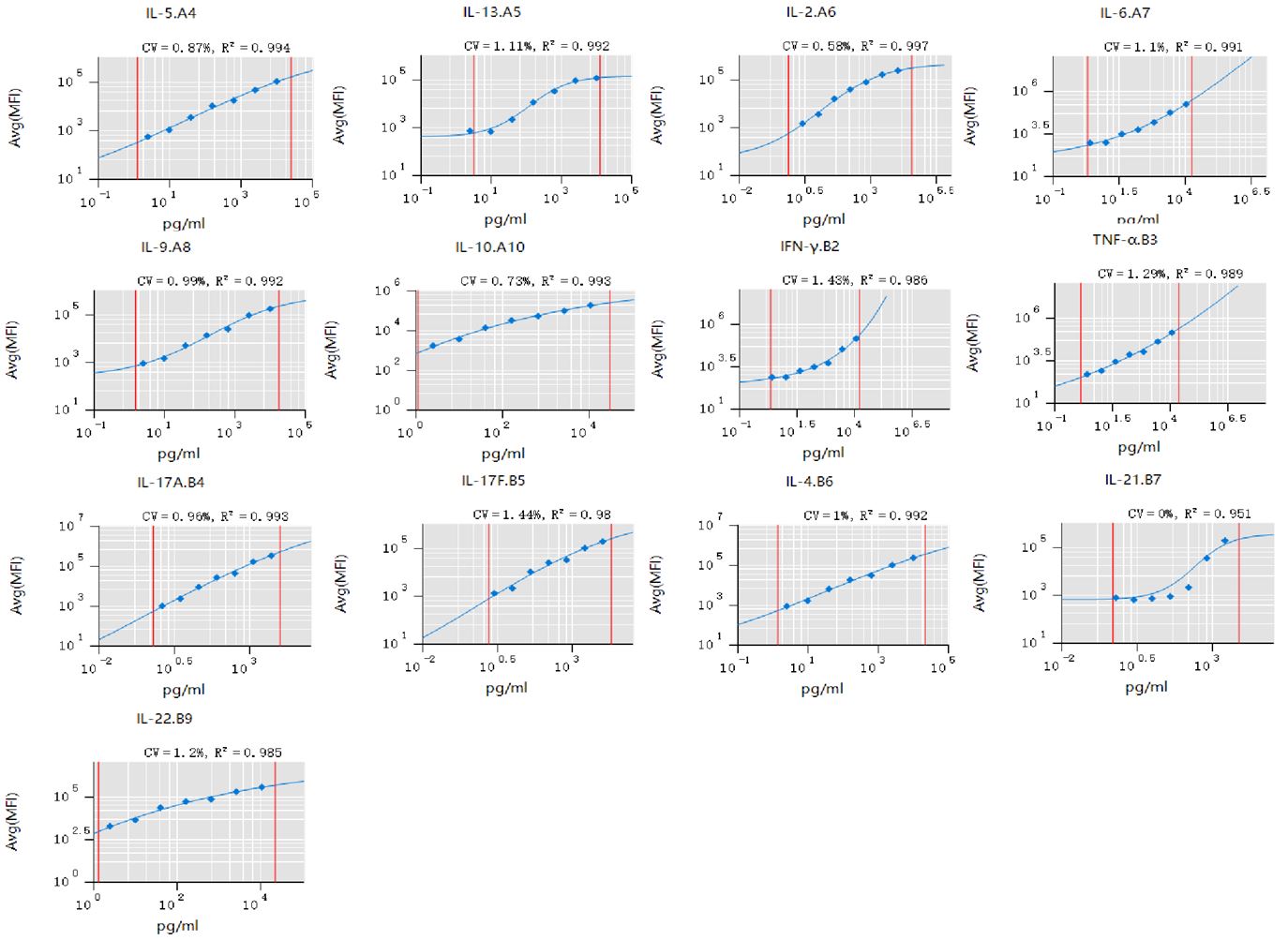


**Supplemental Figure 2. Standard Curves of 13 cytokines by flow cytometry.**


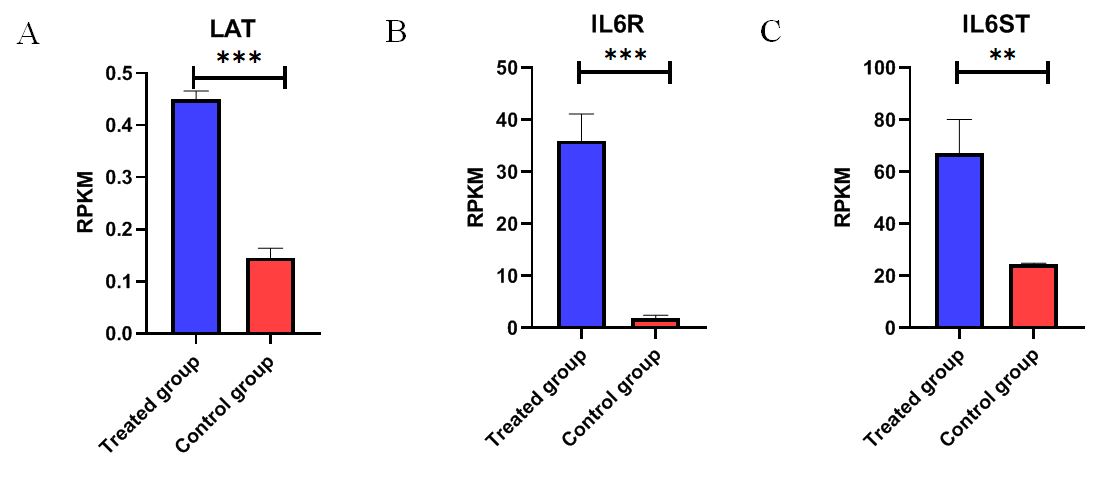


**Supplemental Figure 3. The expression of LAT, IL6R and IL6ST detected by RNA sequencing were significantly elevated compared with pre-treatment.**
